# Patterns of genetic variation and life history traits of *Zeuxapta seriolae* infesting *Seriola lalandi* across the coastal and oceanic areas in the southeastern Pacific Ocean: potential implications for aquaculture

**DOI:** 10.1186/s13071-015-0892-4

**Published:** 2015-05-22

**Authors:** Fabiola A Sepúlveda, M Teresa González

**Affiliations:** Programa Ciencias Aplicadas, mención Sistemas Marinos Costeros, Universidad de Antofagasta, P.O. Box 170, Antofagasta, 1240000 Chile; Instituto de Ciencias Naturales “Alexander von Humboldt”, Facultad de Ciencias del Mar y Recursos Biológicos, Universidad de Antofagasta, Antofagasta, 1240000 Chile

**Keywords:** Biological traits, COI, Fish, Genetic diversity, Monogenea, Genetic structure

## Abstract

**Background:**

The monogenean, *Zeuxapta seriolae*, is a host-specific parasite that has an extensive geographical distribution on its host, *Seriola lalandi*, and is considered highly pathogenic in farmed fish. In recent years, developing cultures of *S. lalandi* in different coastal localities in Southeastern Pacific Ocean (SEP) have been affected by moderate and heavy infections of this parasite, attributed to contagion from wild to farmed fish. Here, we evaluated the pattern of genetic variations and biological traits of *Z. seriolae* in a spatial and temporal scale across its geographical distribution in SEP to determine its genetic status and biological traits, which could affect its transmission dynamics from wild to farmed fish.

**Methods:**

Wild fish and their parasites were sampled from fisheries in the northern Chilean coast (NCC: 24°S-30°S) and Eastern islands (JFA: ca 33°S; 80°W) between 2012 and 2014. Fragments of 816 bp of the cytochrome *c* oxidase subunit I (COI) gene was sequenced for 112 individuals from NCC and 63 from JFA and compared using AMOVA. Prevalence and intensity of *Z. seriolae* were calculated for each area. The parasite body size, fecundity and size at sexual maturity were estimated for 177 parasites from NCC and 128 from JFA, and significant differences were evaluated using GLM.

**Results:**

Geographical genetic structuring was detected for *Z. seriolae* across SEP, with a population in NCC and the other in JFA, both with the same high haplotype diversity. Neutrality tests and mismatch analyses indicated that both *Z. seriolae* populations are stable. Parasite biological traits such as fecundity, body size, and size at sexual maturity, and population parameters varied significantly between geographical areas.

**Conclusion:**

Two genetic groups of *Z. seriolae* were detected in wild fish across SEP. Because of the seasonal migration of wild host and temporal contact with farming, quantifying the genetic diversity and level of gene flow or isolation between parasite populations is useful for fish health management in farming. The smallest size of sexual maturity in parasites from NCC is predictive of shorter life cycles, and their high genetic diversity suggests high evolutionary potential and high transmission of this parasite to farmed hosts.

## Background

Patterns of genetic diversity or variations among parasite populations can provide clues to the population life histories and degree of evolutionary isolation, which may have important applications in aquaculture and epidemiology [1, 2]. Therefore, the population genetic structure of a parasite, which is associated with the amount of genetic exchange between subpopulations, allows for understanding the parasite’s dispersal capabilities and identifying a source of infection on a spatial scale, which contributes to the control of diseases [2]. The extent of geographic variations in gene frequency in most species is the result of the geographic distance between subpopulations and is associated with variations in the oceanographical conditions [3–5]. Further, balances among mutation, genetic drift, selection and genetic flow can produce either local genetic differentiation or genetic homogeneity [6]. Of these aspects, gene flow, which includes all mechanisms that result in the movement of genes from one population to another, determines the extent to which each local population of a species is an independent evolutionary unit [7, 8]. For parasites, genetic flow among populations also depends on the intrinsic host-parasite relationship [9, 10]. Therefore, high or low genetic flow in parasite populations has important implications for evolutionary processes, such as host-race formation, adaptation to host defenses, and the evolution of drug resistance [11]. Moreover, it has been hypothesized that a pathogen population with high evolutionary potential, which is the potential to generate new variations in traits that determine host-parasite interactions and the evolution of disease dynamics [12], is associated with high mutation rates, high potential for gene flow and a large population effective size (N_e_), each of which allows for the increase of genetic diversity and presents a higher risk of breaking down resistance genes [13].

The genetic structure of parasite populations is associated with host specificity, host mobility and environmental conditions [9]. The dispersal ability of parasites with low host specificity and in the absence of physical barriers greatly facilitates extensive gene exchange among different subpopulations [12, 14, 15]. Alternatively, host specific parasites are more likely to experience frequent local extinction and re-colonization events, particularly in small and fragmented wild host populations. These population processes may promote the loss of genetic diversity within a parasite population and generate genetic differences among populations through genetic drift [12]. Additionally, the potential spatial distribution of a parasite not only depends on the dispersal stages of parasites (the free living stages, e.g., eggs, oncomiracidium larvae, and nauplius larvae) but is also closely coupled with host mobility (sedentary or highly mobile), thereby facilitating the homogenization of a parasite population and allowing for the potential evolution and spread of drug resistance in parasites [16], as demonstrated for terrestrial nematodes [11] and marine hosts [17], freshwater trematodes [16] and marine monogeneans [15].

In marine systems, oceanographic conditions and the distance between populations can determine the geographic genetic patterns in parasites [18, 19] and, similarly, differences in parasite biological traits. Environmental parameters, such as temperature and salinity in different latitudes, may affect the egg production rate, egg-hatching time and development to sexual maturity in marine ectoparasites because these occur more quickly at warmer temperatures and can trigger different infection dynamics [20–22].

Population genetic studies provide insight into parasite evolutionary histories and aid in the identification of the causal factors contributing to disease dynamics and distribution [12]. The monogenean, *Zeuxapta seriolae*, is reported in *Seriola lalandi* from Australia, New Zealand, Japan and California and in *S. dumerili* from the Mediterranean Sea [23–26]. The genus *Zeuxapta* is specific to the genus *Seriola,* and *Z. seriolae*, and shows a particularly wide geographic distribution in both farmed and wild fish. Additionally, *Z. seriolae* is considered highly pathogenic in farmed fish because at high intensities, it can kill its host by causing anaemia [25, 27–30].

The yellowtail kingfish *S. lalandi* is a pelagic fish and is highly migratory and widely distributed in temperate and subtropical waters of the world. In the SEP, *S. lalandi* present a permanent population in an archipelago approximately 700 km from continental Chile (80°W), where it is captured year-round. On the northern continental Chilean coast (NCC: 20°S to 30°S), this species arrives annually in the summer, most likely to feed, as occurs in the southwest Atlantic Ocean [31]. No spawning and nursery areas are known in SEP. In recent years, cultures of *S. lalandi* have been initiated at different localities in the NCC, where farmed fish are affected by moderate and heavy infection levels of this parasite, which has been attributed to contagion from wild to farmed fish.

Because *Z. seriolae* is a host specific parasite that has an extensive geographic distribution (cosmopolitan) and affects the aquaculture of *S. lalandi* worldwide it is important to know its evolutionary potential, which can be useful for understanding the dynamic of the disease. Here, we evaluated the patterns of genetic variation of this monogenean on spatial and temporal scales using mitochondrial DNA markers. Additionally, we estimated several life history traits, such as fecundity and size at sexual maturity, beside population parameters of infection across the geographic distribution in the SEP. Taking into account that the host population migrates in the summer to the coast in the SEP (extending the fishery from 20 °S to 30 °S) but the host migratory route, fish origin and the effect of physical barriers on the dispersal of parasite population are not known, we can expect that parasite populations correspond to a single panmictic population. Alternatively, if physical barriers or host segregation affect the genetic flow of parasites, we can expect genetic structure of *Z. seriolae* across the SEP.

## Methods

### Sampling areas and collection of parasites

**Fig. 1:**
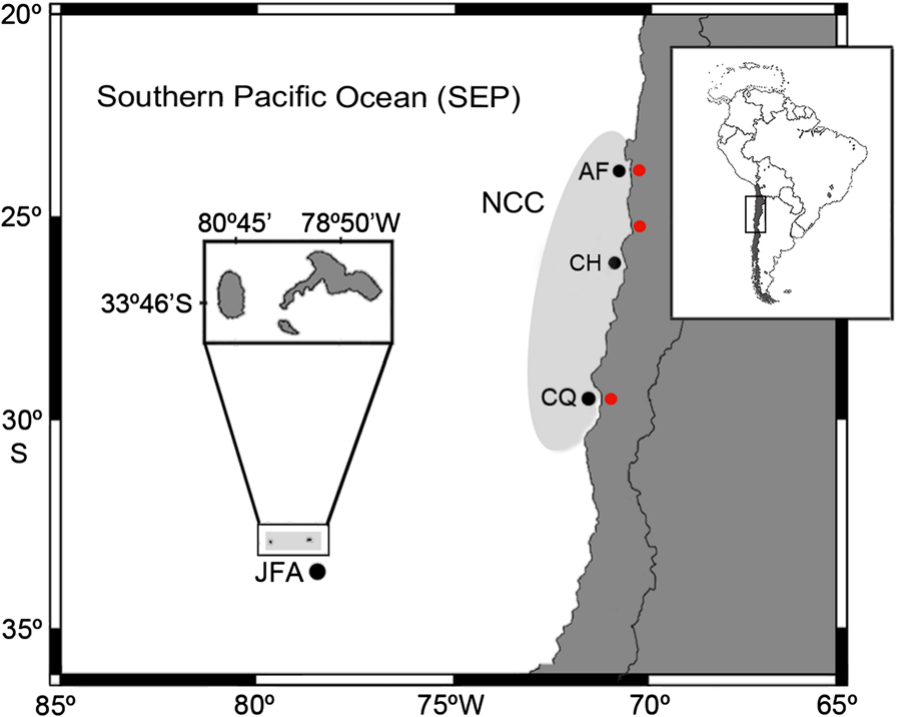
Area of study showing sampled localities in the Southern Pacific Ocean (SEP). Northern Chilean coast (NCC): AF, Antofagasta; CH, Chañaral; CQ, Coquimbo. Oceanic area: Juan Fernández Archipelago (JFA). Black circle: fishery locality; red circle: farming locality

Parasites were collected off 393 wild *S. lalandi* fish captured by commercial fishers from two geographical areas (coastal and oceanic) across the SEP. Fish from the oceanic area (JFA: 33 °S, 80 °W) were collected between April and August of 2013–2014. Fish from the coastal area were collected from different sites during summer (January and April) between 2012 and 2014 because fishing of *S. lalandi* extends from 20 °S to 30 °S varying the fish landing sites between years. The sampled sites/year in the NCC were: Antofagasta/2013, 2014 (AF: 24 °S, 70 °W), Chañaral/2012 (CH: 26 °S, 70 °W) and Coquimbo/2014 (CQ: 30 °S, 71 °W) (Fig. 1).

For molecular analyses, only one parasite per fish was used in 123 fish to prevent the sampling of inbred offspring [14]. Two parasites per fish were collected from 23 fish to increase the number of parasites by locality and year. In only two cases, three parasites from one fish were used to prior check that they were not clones. Specimens of monogenean *Z. seriolae* were removed from the gills of fish, counted and identified using a stereomicroscope, and each parasite was placed individually into a 1.5 ml Eppendorf tube with absolute ethanol for DNA extraction.

### DNA extraction, amplification and sequencing

DNA was extracted using the QIAamp Tissue kit (Qiagen, Germany) according to the manufacturer’s instructions. The DNA was then eluted into 50 μl of nuclease free water. Mitochondrial gene cytochrome *c* oxidase 1 (COI) was amplified using forward primer JB3 (5′-TTTTTTGGGCATCCTGAGGTTTAT-3′) and reverse primer COX1 (5′AATCATGATGCAAAAGGTA-3′) as described by Leung *et al*. [32]. Each PCR reaction had a final volume of 35 μl including: 5 units of GoTaq® DNA polymerase (Promega), 7 μl 5X PCR buffer, 5.6 μl MgCl2 (25 mM), 2.1 μl BSA (10 mg/ml), 0.7 μl of deoxynucleotide triphosphate (dNTP) (10 mM), 10 pM of each primer and 5 μl template DNA. The thermocycling programme included: an initial denaturation step at 95 °C for 2 min followed by 40 cycles of amplification at 95 °C for 30 s, 48 °C for 40 s and 72 °C for 1 min, with a final extension step at 72 °C for 10 min. PCR products were purified using E.Z.N.A® Cycle-Pure PCR Purification Kit (Omega Bio-Tek, Inc., Atlanta, Georgia, USA), and both DNA strands were directly sequenced (Macrogen, Seoul, Korea; http://www.macrogen.com).

### Data analyses

Sequences were edited using ProSeq v 2.9 beta [33] and aligned using Clustal 2 software package [34].

### Genetic diversity and population structure

Molecular diversity was estimated through the following indices: number of haplotypes (H), number of polymorphic sites (S), haplotype diversity (H*d*: a measure of the frequencies and numbers of haplotypes among individuals [35]), nucleotide diversity (π: average weighted sequence divergence between haplotypes [35]) and mean number of pairwise differences (k) that were computed using Dnasp 5.0 [36] and Arlequin v3.1 [37]. Genetic distance within locations and between localities was estimated using Mega 6.0 [38]. Mann Whitney U test were used to evaluate differences in genetic diversity of *Z. seriolae* between geographical areas.

Genetic population structures were examined through AMOVA to determine the amount of genetic variability using F-statistics in three temporally subdivided hierarchical levels: the proportion of variations among years (F_ct_), among populations within years (F_sc_) and within populations (F_st_). The significance of the covariance component associated with different possible levels of genetic structure was permuted 10,000 times. Pairwise genetic differentiation between populations was estimated using the fixation index F_st_ and statistical significance was tested with 10,000 permutations. Both analyses were performed in Arlequin v3.1.

### Demographic analysis

Episodes of population growth or decline show characteristic signatures in the distribution of nucleotides between pairs of individuals that in *Z. seriolae* were evaluated with mismatch distribution analysis in each locality and year and for total samples, with analysis performed using Dnasp 5.0. Unimodal distribution and multimodal distributions were thereby distinguished for historic expansion and population equilibrium, respectively [39].

In addition, two neutrality tests were performed in Arlequin v3.1 software: Tajima’s D [40] and Fu *F* statistics [41]. Both neutrality tests provide predictions about evolution under mutation-drift equilibrium in the absence of systematic effects such as selection or demographic effects. Thus, significant deviations from neutrality can be a consequence of selection, population expansions, bottlenecks or demographic fluctuations [42].

Finally, a haplotype network was constructed using HaploViewer (http://www.cibiv.at/~greg/haploviewer, Center for Integrative Bioinformatics Vienna) previous construction of a neighbour-joining tree (TN93 + G model) in Mega v6. All sequences were deposited in GenBank under accession number: KP119183-KP119357.

### Population parameters, fecundity and body size

Prevalence and intensity of *Z. seriolae* were calculated according to Bush *et al.* [43] for years, sites and geographical areas. The median intensity was used as descriptor of central tendency because data do not show normal distribution [44]. Three hundred and five *Z. seriolae* specimens were randomly selected to estimate parasite body length and fecundity by year, site and geographical area. Each parasite was individually examined in a slide with a drop of water and cover slip. Measurements were carried out using Micrometrics 5.0 software (New York Microscope Company, Inc.), which was connected to an Olympus camera. The total length (in millimeters) included the opisthaptor length.

Parasite fecundity was measured as number of eggs per parasite. For this, the parasite uterus was dissected and an entire chain of eggs was removed and counted using a manual counter. To evaluate differences in fecundity, only those parasites whose uteri contained over 50 eggs were considered to exclude those parasites just starting their egg production.

The size at first sexual maturity (= first size egg production) was measured as the parasite length when 50 % of the population of *Z. seriolae* contained at least one egg within the uterus [45] for each fishing area. For this estimation, all examined parasites (with and without eggs) were used (n_NCC_ = 590; n_JFA_ = 158). The parasite length was categorized in mm (1 mm to 25 mm).

2x2 contingency tables with Yates correction were used to evaluate prevalence between geographical areas and temporal variations for sites AF (coastal) and JF (oceanic). Mann Whitney U tests were used to evaluate differences in intensity of *Z. seriolae* between geographical areas and to compare the parasite total length between the NCC and JFA samples. The Spearman correlation was used to evaluate the association between parasite body length and number of eggs [44]. Generalized linear models were used to evaluate differences in intensity of infection and parasite fecundity between geographical areas. Fish size was used as co-variable for intensity, and total parasite length was used as co-variable for fecundity. For both models, we used the poisson distribution for response variable and the log as the function link [46]. All analyses were performed using Statistica 7.0 software (Statsoft Inc., Tulsa, Oklahoma).

## Results

### Genetic diversity and population structure

A 816 bp segment of the COI gene was obtained from 175 *Z. seriolae* individuals sampled from the two geographical areas: 112 sequences from the NCC and 63 sequences from the JFA (Table 1). Sequence variability between NCC and JFA was 0.5–0.9 %, while intra-locality variability was 0.4–0.8 %.

**Table 1:** Genetic diversity and neutrality test for Zeuxapta seriolae by sampling localities and years

Area	Site-year	N	H	S	H*d*	π	*K*	Tajima’s *D*	Fu’s F_S_
NCC	CH-2012	14	6	12	0.79 ± 0.09	0.004 ± 0.0014	3.28	−0.51	0.34
	AF-2013	41	13	21	0.85 ± 0.04	0.006 ± 0.0006	5.26	0.24	−0.41
	AF-2014	26	15	24	0.89 ± 0.05	0.007 ± 0.0006	5.96	−0.19	−3.25
	CQ-2014	31	17	25	0.91 ± 0.04	0.007 ± 0.0004	6.14	−0.06	−3.84
	TOTAL NCC	112	34	40	0.88 ± 0.02	0.007 ± 0.0003	5.59	−0.80	−11.51*
JFA	JF-2013	36	20	21	0.87 ± 0.05	0.006 ± 0.0007	4.77	−0.19	−8.03*
	JF-2014	27	13	15	0.87 ± 0.04	0.005 ± 0.0007	4.02	0.11	−3.15
	TOTAL JFA	63	31	25	0.88 ± 0.04	0.005 ± 0.0005	4.48	−0.49	−18.55**
TOTAL		175	61	48	0.91 ± 0.01	0.007 ± 0.0001	5.70	−0.99	−34.35*

N, number of sequences analyzed; H, number of haplotypes; S, number of segregating sites; H*d*, haplotype diversity (±s.d.); π, nucleotide diversity (±s.d); *K*, mean pairwise difference; Tajima’s *D* test and Fu’s FS test . *Significant *p*-values < 0.05; **p-values < 0.001. Codes for localities are given in Fig. 1

**Fig. 2:**
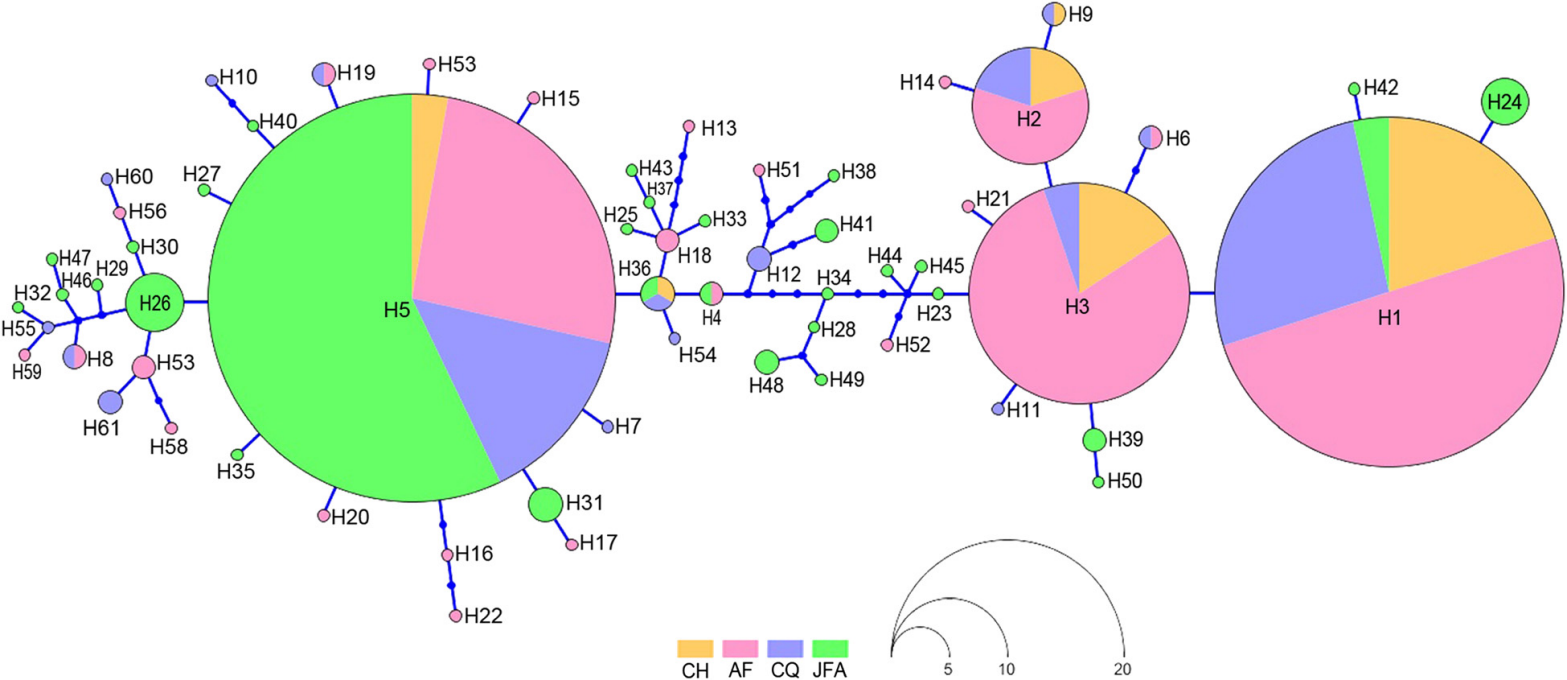
Neighbour-joining network for mtDNA COI gene haplotypes of *Zeuxapta seriolae*. The size of the circles is proportional to the haplotype frequency, and the colour represents the population to which they belong. Locality codes are shown in Fig. 1

A total of 48 polymorphic sites led to the definition of 61 haplotypes (Table 1), 27 of which were unique to the JFA (44.3 %), whereas another 10 haplotypes (H1, H2, H3, H4, H5, H6, H8, H9, H19, H36) were shared among the different areas (NCC and JFA, 16.4 %), and 24 haplotypes were unique to the NCC (39.3 %). Among the shared haplotypes, H1 and H5 were the most frequent haplotypes, with 90 % of H1 predominant in the NCC and 58 % of H5 predominant in the JFA (Fig. 2).

Genetic diversity indices of *Z. seriolae* within and between geographic areas are summarized in Table 1. Total haplotype diversity considering the entire region of study was 0.91 ± 0.01, and nucleotide diversity was 0.007 ± 0.0001. The haplotype diversity did not differ between geographical areas (U = 37.5; *p* = 0.42), but nucleotide diversity was significantly lower in JFA (U = 9; *p* = 0.002; Table 1).

Hierarchical AMOVA analysis revealed significant genetic differentiation at the spatial scale (F_st_ = 0.1286, p < 0.05; F_sc_ = 0.1354, p < 0.05). Eighty-seven percent of the variance was explained by variability within populations and 13 % of the remaining genetic variation was attributed to variability among populations within years (Table 2). Parasites from JFA were significantly different from those from the NCC (Table 3); therefore, JFA is considered as a different population from NCC. At temporal scale, there were no significant genetic differences for parasites from JFA between years, but parasites from NCC showed differences between site CH of year 2012 compared with AF and CQ of 2014 (Table 3).

**Table 2:** Summary of hierarchical molecular variances analysis for Zeuxapta seriolae

Source of variation	*d.f.*	Sum of squares	Variance components	Percentage of variation	F statistics	*P* value
Among years	2	22.338	−0.0229	−0.79	F_ct_ = −0.0079	0.4484 ± 0.0052
Among populations within years	3	45.048	0.3975	13.65	F_sc_ = 0.1354	0.0054 ± 0.0008
Within populations	169	428.928	2.5380	87.14	F_st_ = 0.1286	<<0.05
Total	174	496.314	2.9726

**Table 3:** Pairwise F_ST_ and p-values for test of population differentiation for Zeuxapta seriolae across Southeastern Pacific

G.A.	Locality-year	CH-2012	AF-2013	AF-2014	CQ-2014	JF-2013	JF-2014
NCC	CH-2012		0.03821	0.1193	0.1745	0.4139	0.4702
	AF-2013	0.15 ± 0.01		0.0048	0.0259	0.2043	0.2367
	AF-2014	0.035 ± 0.006	0.27 ± 0.014		−0.0050	0.1261	0.1562
	CQ-2014	0.017 ± 0.004	0.13 ± 0.01	0.37 ± 0.016		0.0521	0.0881
JFA	JF-2013	<<0.001	<<0.001	0.0068 ± 0.003	0.044 ± 0.006		0.0144
	JF-2014	<<0.001	<<0.001	0.0039 ± 0.002	0.030 ± 0.005	0.19 ± 0.01	

F_ST_ above diagonal; p-value below diagonal. Geographical Area: G.A.; Northern Chilean Coast (NCC): CH, AF, CQ; Juan Fernández Archipelago (JFA): JF

### Demographic history

The *Z. seriolae* haplotype network revealed the existence of two genetic groups: the first group showed one main ancestral haplotype (H5) occurring in each site and geographical area, but it was more predominant in JFA where H5 was surrounded by a large number of unique haplotypes. The group 2 included three highly frequent haplotypes (H1, H2, H3) that were predominant in the NCC (Fig. 2).

**Fig. 3:**
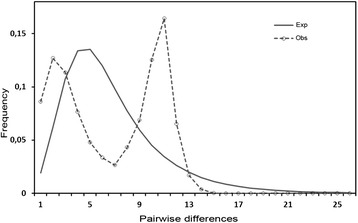
Mismatch distributions of COI gene haplotypes for total samples of *Zeuxapta seriolae*. The graph was constructed using pairwise differences observed and expected under the expansion model

The mismatch distribution for each site (graph not shown) and for entire geographical area of study deviated significantly from the expected distribution under expansion model, exhibiting a bimodal distribution of pairwise differences (Fig. 3). Neutrality test showed lack of significance of the Tajimas *D* test (*D* = −0.996, p > 0.1; Table 1), indicating that parasite populations are in equilibrium under the neutral model. Fu’s test, however, was significantly negative (Table 1) for NCC and JFA parasite populations, suggesting the influence of some process affecting the demographical history of *Z. seriolae*.

### Population parameters, fecundity and body size

Prevalence (χ^2^ = 31.7; df = 1; p < 0.001) and intensity (U_193,78_ = 5173.5; p < 0.001) of *Z. seriolae* were significantly higher in JFA, considering the entire period of study (Table 4). In the NCC, the prevalence varied between 52.3 % in year 2013 and 72.5 % in year 2014. In JFA, the prevalence varied between 95 and 97 % in years 2013 and 2014 respectively. Prevalence varied between years within site AF in the coastal area (χ^2^ = 4.96; df = 1; *p* = 0.02) but not in the oceanic area (χ^2^ = 0.115; df = 1; *p* = 0.73). Among years, median intensity varied between 2 and 14 in the NCC and between 8 and 15 in the JFA (Table 4). A GLM showed that differences in intensity were affected for area (χ^2^ = 41.6; p < 0.001), year (χ^2^ = 5.1; *p* = 0.02) and co-variable fish size (χ^2^ = 101.9; p < 0.001).

**Table 4:** Population parameters and biological traits for Zeuxapta seriolae across the Southern Pacific

Area	Site-year	N_H_	P%	I (MI)	N_P_	PBL (± sd)	F (MF)
NCC	CH-2012	54	70.37	1-100 (6.5)	72	12.82 (2.2)	50-1112 (135)
	AF-2013	154	52.38	1-91 (2)	68	14.12 (3.2)	51-789 (216)
	AF2014	66	68.18	1-131 (4)	14	19.9 (3.9)	64-862 (305)
	CQ-2014	39	72.5	1-108 (14)	23	17.77 (3.5)	60-1347 (282)
	**TOTAL NCC**	313	61.98	1-131 (3)	177	14.56 (3.7)	50-1347 (189)
JFA	JF-2013	20	95	5-55 (15)	105	15.3 (2.4)	54-1085 (265)
	JF-2014	60	96.67	1-25 (8)	23	13.49 (2.0)	53-612 (259)
	**TOTAL JFA**	80	96.25	1-55 (10)	128	14.98 (2.4)	53-1085 (259)

Prevalence (P%), intensity range (I), mean parasite body length (PBL), fecundity range (F) per site, year and total period of study*.* MI, median intensity; MF, median fecundity; N_H_, number of examined hosts; N_P_, number of examined parasites; s.d, standard deviation

Fecundity correlated with parasite body length (r_s_ = 0.156; *n* = 341; *p* = 0.003). *Z. seriolae* fecundity variations were explained by geographical area (χ^2^ = 343.06; p < 0.001) and mostly by the co-variable parasite body length (χ^2^ = 7743.03; p < 0.001).

**Fig. 4:**
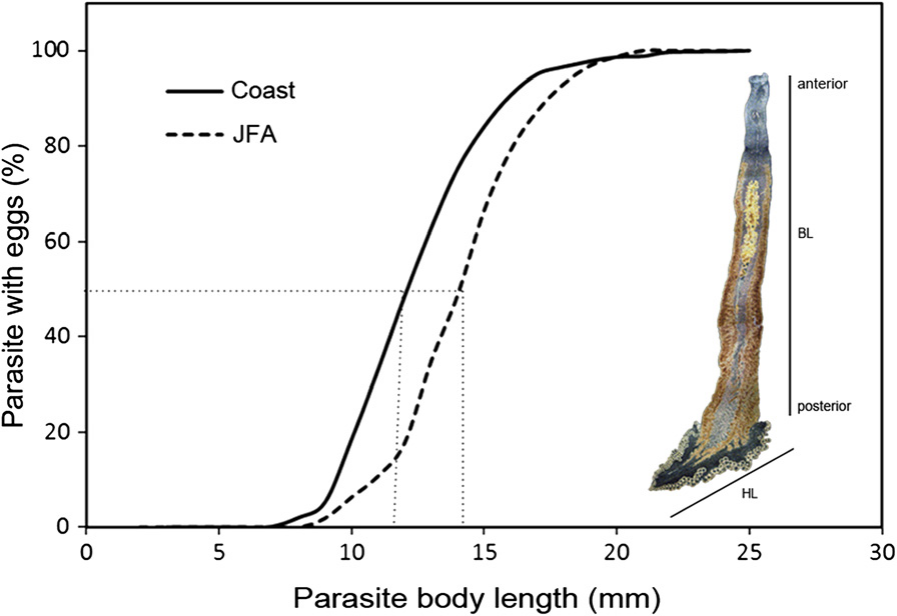
First size of sexual maturity of *Zeuxapta seriol*ae by geographical area. HL: haptor length and BL: body length

Parasites from the JFA were significantly longer than those from the NCC (Table 4). The range of total *Z. seriolae* body length varied from 3.06 to 24.31 mm in the NCC and from 1.65 to 25.8 mm in the JFA. The first size at sexual maturity of *Z. seriolae* reached 11.8 mm in NCC, and at 14.4 mm in JFA (Fig. 4). Consequently, parasites from the JFA reached sexual maturity at higher body lengths.

## Discussion

For the effective management and health maximization of cultured fish, it is essential to have a clear understanding of the levels of gene flow, the connectivity of parasite populations or metapopulations, the sources of larval infestations, and the relative fluxes of parasites between wild and farmed fish [1, 2]. To study these factors, genetic markers, such as mtDNA, have been widely used to detect population genetic structures [47, 48], which is useful for estimating the evolutionary potential of a parasite. Our results using the COI gene provide a geographical genetic structure for the monogenean *Zeuxapta seriolae*, with one population present in their host *S. lalandi* that approaches the coast in the summer and the other population in host fish from the oceanic area in the southeastern Pacific Ocean (SEP). This parasite population genetic structure may be explained by oceanographical barriers to parasite dispersion and/or by host population segregation.

Population structure in space and time is the result of both present processes and past history [49]. Populations that are established from small numbers of individuals tend to lose genetic variation due to increased effects of genetic drift [50]. However, successful colonization occurring during a long time period can accumulate new mutations, and species with relatively quick generation turnover, high fecundity and short lifespan like parasites can favour a fast rate of molecular evolution [51]. In our study, the bimodal mismatch distribution, which depends on the evolutionary history of one population [39], suggest that *Z. seriolae* populations are stable and consistent with populations that are geographically subdivided and have limited or low migration [52]. Additionally, network haplotype also suggests stable populations and are consistent with the lack of significance showed for neutrality Tajima’s test. This test is based on the allele frequency distribution of segregating nucleotide sites [40], while Fu’s test uses the distribution of alleles or haplotypes [41]; this last being considered the most sensitive test to detect some selection (or expansion) process [53]. Thus, the significant values in Fu’s test indicate an excess of rare haplotypes what would be expected under neutrality (H5 connected to a high number of unique haplotypes), suggesting that in the past some purifying selection process on *Z. seriolae* populations [41, 54] has taken place, which could have occurred as a consequence of two possible scenarios: when host fish colonized oceanic Eastern island, therefore, introducing the parasites [55] or when parasites infecting migrant fish colonized fish from JFA. Likewise, the lower genetic diversity in parasite populations from JFA might be associated with ‘founder effect’ [56, 57] as has been suggested for the monogenean *Mazocraeoides gonialosae* along the coast of China [14].

Parasites infecting highly mobile hosts such as *S. lalandi*, could reach remote locations as well as its host. However, geographical barriers can affect either the host or parasite distribution [20, 58]. JFA corresponds to islands of volcanic origin along hotspot lines of the Nazca Plate and emerged in the Plio-Pleistocene period, approximately 4 million years ago, [59]. These islands are located between the coastal and oceanic branches of the sub-Antarctic Peru or Humboldt Current, which is split by the subtropical Peruvian countercurrent [60]. Studies of the zoogeography of icthyofauna suggest that the JFA and other groups of oceanic islands in the SEP should be considered a biogeographic unit [61, 62]. Until now, previous studies of other carangids, such as *Trachurus murphyi* [63], have not demonstrated that oceanographic barriers limit their dispersion and distribution in SEP*.* However, factors such as the Chile–Peru Current (Humboldt Current), surface gradients of temperature and salinity, and the great depths of the Chile–Peru Trench (more than 4000 m in the region) [64, 65] may impose barriers to the dispersion of parasites and may thus explain the genetic structure detected in the *Z. seriolae* population there.

The dispersal ability and genetic flow of ectoparasites is influenced by host specificity [66]. Host specificity is a key property of parasites because it is a determinant of their local extinction risk and their likelihood for successful establishment following introduction to a new region, with generalist species less prone to local extinction and better invaders than specialists [67]. The monogeneans *M. gonialosae* and *Gotocotyla sawara*, which are considered generalist parasites that infect more than one host fish species, did not show a geographical genetic structure based on COI mtDNA in the western Pacific Ocean. The absence of different genetic structures between the populations of these parasite species was attributed to a high gene flow favoured by their host range [14, 15]. The dispersion stages of *Z. seriolae* consist of a string of eggs that can entangle in the gills of fish or join together to form light masses of numerous eggs that have a wide surface area, which allows the eggs to stay in the water column for a short time [28], with passive dispersion and free-swimming larvae that live approximately 24 h (personal obs.). The short lifetime of these developmental stages in the water column, their passive mode of dispersion and their high host specificity may decrease the probability of encountering a suitable host that favours their dispersal and genetic flow across an extensive geographical area. These parasite characteristics suggest a low degree of parasite migration between host populations, which may contribute to local adaptations [68].

Host behaviour is an important factor for parasite dispersal [9]. Therefore, hosts with an interrupted geographical distribution will affect the gene flow of their parasites [69]. *S. lalandi* is a cosmopolitan species distributed in the Pacific, Indian and Atlantic Oceans (www.fishbase.org). This species arrives at the NCC annually between December and April, where it is captured by artisanal fishermen, whereas *S. lalandi* is captured in the JFA year-round. In other geographical areas, *S. lalandi* shows a reproductive strategy with limited migration; specifically, only a subset of the reproductive fish migrates [70]. Additionally, tagging studies conducted in Australia suggest that *S. lalandi* can migrate considerable distances, but most juveniles are relatively sedentary [23, 71]. However, spawning and nursery areas of *S. lalandi* are unknown in the SEP, the geographical genetic differences between *Z. seriolae* from JFA and NCC as well as the temporal genetic differentiation of *Z. seriolae* between localities from NCC could be indicative of different host subpopulations. Alternatively, the short generation time of the parasites combined with the subdivision of the host populations during prolonged time periods may contribute to the generation of structured parasite populations.

Throughout a latitudinal gradient, variations in environmental parameters, such as temperature and salinity, can modify the biological traits of a species [72, 73]. Monogenean biological traits vary according to environmental parameters, such as temperature and salinity, resulting in individuals achieving sexual maturity more quickly at warmer temperatures [20, 74–76]. *Z. seriolae* from the NCC area showed a smaller first size at sexual maturity and was significantly smaller and produced fewer eggs than did parasites from the JFA. Tubbs *et al.* [20] suggested an optimal temperature of 17.5 °C for the *in vitro* fecundity of *Z. seriolae* because egg production decreases at other temperatures yet sexual maturity is reached more quickly at higher temperatures. The seawater in NCC varies between 18 °C and 20 °C in summer [77] and between 14 °C (winter-autumn) and 20 °C (spring-summer) in the waters of JFA [78]. Therefore, it is possible that the local environmental conditions can modify the biological traits of *Z. seriolae* populations across the SEP; however, it is also possible that genetic differences among the parasite populations are reflected in different biological traits. Regardless, the biological trait differences between populations of *Z. seriolae* may involve differential infestation dynamics (i.e. duration of parasite life cycles, infestation rates and parasite loads) on host populations from different geographical areas, as demonstrated for different ectoparasites in fish farming [20, 79, 80]. Similarly, the lower first size at sexual maturity in the NCC is predictive of shorter life cycles of this parasite in this area and probably quickly re-infests, which is associated with high densities of fish in captivity increasing the risk of outbreaks of this disease in farming [81, 82].

## Conclusions

In this study, we detected two different populations of the parasite *Z. seriolae* infesting the wild fish *S. lalandi* across the SEP. In each area, the parasites showed different biological characteristics, such as fecundity, body size, and size at first sexual maturity, and population parameters suggesting different dynamics of infestation. Because of the seasonal migration of wild hosts in the SEP, and consequent temporal contact with farmed hosts in NCC, the quantification of diversity and genetic differentiation and level of gene flow (or isolation) between parasite populations, as well as potential parasite adaptations, is useful for fish health management in farming. The smallest sizes of sexual maturity observed in parasites from the NCC predict shorter life cycles, which along with the high genetic diversity suggest high evolutionary potential and high transmission of this parasite to farmed hosts.

## Abbreviations

COI: Cytochrome *c* oxidase I
NCC: Northern Chilean coast
JFA: Juan Fernandez Archipelago
SEP: South-eastern Pacific Ocean
AF: Antofagasta
CH: Chañaral
CQ: Coquimbo
